# The Role of the Gut-Liver Axis in Metabolic Dysfunction-Associated Fatty Liver Disease

**DOI:** 10.3389/fimmu.2021.660179

**Published:** 2021-04-16

**Authors:** Rosa Martín-Mateos, Agustín Albillos

**Affiliations:** Department of Gastroenterology and Hepatology, Hospital Universitario Ramón y Cajal, Universidad de Alcalá, Instituto Ramón y Cajal de Investigación Sanitaria (IRYCIS), Centro de Investigación Biomédica en Red de Enfermedades Hepáticas y Digestivas (CIBERehd), Instituto de Salud Carlos III, Madrid, Spain

**Keywords:** gut abnormalities, steatosis, intestinal immunity, intestinal microbiota, metabolic liver disease

## Abstract

The complex interplay between the gut microbiota, the intestinal barrier, the immune system and the liver is strongly influenced by environmental and genetic factors that can disrupt the homeostasis leading to disease. Among the modulable factors, diet has been identified as a key regulator of microbiota composition in patients with metabolic syndrome and related diseases, including the metabolic dysfunction-associated fatty liver disease (MAFLD). The altered microbiota disrupts the intestinal barrier at different levels inducing functional and structural changes at the mucus lining, the intercellular junctions on the epithelial layer, or at the recently characterized vascular barrier. Barrier disruption leads to an increased gut permeability to bacteria and derived products which challenge the immune system and promote inflammation. All these alterations contribute to the pathogenesis of MAFLD, and thus, therapeutic approaches targeting the gut-liver-axis are increasingly being explored. In addition, the specific changes induced in the intestinal flora may allow to characterize distinctive microbial signatures for non-invasive diagnosis, severity stratification and disease monitoring.

## Introduction

Metabolic dysfunction-associated fatty liver disease (MAFLD), previously known as non-alcoholic fatty liver disease (NAFLD), is a major cause of liver-related morbimortality, with an estimated global prevalence of 25.24% (1). The burden of MAFLD is expected to increase paralleling the growing incidence of obesity and diabetes mellitus (2). MAFLD represents the hepatic manifestation of an underlying multisystemic metabolic dysfunction, and, thus, patients are not only at risk of liver-related complications (cirrhosis and/or hepatocellular carcinoma), but also cardiometabolic-related events, which are the leading cause of mortality (1). The term reflects the wide spectrum of the disease, which ranges from simple steatosis, to liver inflammation (steatohepatitis), fibrosis and cirrhosis. The extent of fibrosis is the strongest predictor of overall and liver-specific mortality (3).

Recently, the diagnostic criteria for MAFLD have been reformulated, and now are based on the evidence of hepatic steatosis (demonstrated by biopsy, imaging or validated blood biomarkers), in addition to one of the following criteria: overweight/obesity, type 2 diabetes mellitus, or metabolic dysregulation defined by the presence of at least two metabolic risk factors (4). Therefore, currently it is not mandatory to exclude alcohol consumption or other concomitant liver diseases to make a positive diagnosis of MAFLD, as it can frequently coexist with other conditions.

The interaction between the gut, its microbiome and the liver, is key to the pathogenesis of MAFLD, and thus, a significant number of reviews addressing the role of the gut-liver axis have been published over the past few years (5–7). This evolving field has recently achieved promising therapeutic advances based on microbiota composition manipulation. In addition, the characterization of distinctive microbiota signatures for disease diagnosis and stratification is increasingly been explored. In light of the new evidence, this review aims to summarize the characteristic alterations at the different components of the gut-liver axis, their contribution to the pathogenesis of MAFLD, and the most recent and clinically relevant advances involving the gut-liver axis as a potential therapeutic target and non-invasive diagnostic tool in patients with MAFLD.

## The Role of the Gut-Liver Axis in Liver Disease

The term gut-liver-axis has been coined to highlight the close interaction between the intestine and the liver, which also involves a complex interplay with the gut microbiome and the immune system (8). The portal vein serves as the main functional link leading nutrients and gut-derived antigens toward the liver, which in turn provides bile acids, proteins, lipids and immune components. Although highly specialized epithelial and vascular barriers regulate the transport across the intestinal mucosa, a wide variety of microbial products and bacteria reach the liver through the portal circulation. Most of them are harmless dietary and commensal products, and thus, the immune system cells involved in surveillance remain tolerant upon recognition. However, when gut-derived bacterial pathogens reach the liver sinusoids, an effective immune response is activated in order to prevent their spread through the systemic circulation. The disruption of the gut-liver axis alters the balance between immune activation and tolerance (9), and the subsequent immune dysfunction critically contributes to the pathogenesis and progression of liver diseases, and in particular, to MAFLD (5).

### Gut Microbiota as a Key Regulator of the Metabolic Profile

The gut microbiota comprises a diverse spectrum of microorganisms involved in metabolic, synthetic and regulatory functions, including fermentation of non-digestible dietary substrates, synthesis of vitamins, bile acids metabolism, regulation of epithelial cell proliferation or modulation of the inflammatory response (10). The gut microbiota also hinders pathogen colonization by competing for nutrients and space, and thus, it represents the first-line defense of the intestinal barrier.

Microbiota composition is critically influenced by genetic and environmental factors, such as diet, alcohol consumption, or certain drugs. These factors lead to quantitative and qualitative alterations that significantly impact the metabolic activity of the bacterial community and are involved in the pathogenesis of multiple diseases (11–13). In particular, dysbiosis and the alteration of the intestinal barrier are strongly associated with inflammation and metabolic disorders (14). Preclinical studies have shown that microbiome from obese mice has an increased capacity to harvest energy from the diet and also influences how this energy is used and stored (15). In humans, low bacterial richness and distinctive alterations of the metagenome allow to define subsets of individuals with high-risk metabolic profiles (16), and, also, identify specific microbiome signatures for related diseases such as type-2 diabetes (17) or obesity (18).

#### The Impact of Gut Microbiome in the Pathogenesis of MAFLD

Consistent with the growing evidence that suggest an strong correlation between the microbiome and the metabolic dysfunction, the role of the microbiota in the pathogenesis of MAFLD has also been explored (19). In this regard, germ-free mice have shown a significant decreased susceptibility to diet induced hepatic steatosis (20). This pathogenic role is further highlighted by the fact that microbiota composition can determine the response to the administration of high-fat diet, leading to hyperglycemia and hepatic steatosis depending on the bacterial host profile. Interestingly, the propensity to develop MAFLD in response to high-fat diet may be transmissible by gut microbiota transplantation (21).

Taxonomic studies have proposed distinctive microbiome signatures based on the flora modifications associated to the different stages of the disease (22). On one hand, some authors have shown that, compared with healthy subjects, the Proteobacteria and Fusobacteria phyla are more abundant in patients with MAFLD (23). Conversely, a lower abundance of Bacteroidetes, and in particular, the genera *Prevotella*, has been found in this group. With regards to steatohepatitis, increased abundance of *Bacteroides* and decreased concentrations of *Prevotella* were independently associated with liver inflammation in a well-characterized population of adult MAFLD (24). These quantitative changes were accompanied by significant shifts in the modulatory functions of the microbiota and the metabolism of carbohydrates, lipids, and amino acids (24).

As MAFLD progresses from liver steatosis and inflammation to fibrosis, other distinctive signatures have been found. Metagenomic sequencing has identified an association between advanced fibrosis and an increased abundance of *Escherichia coli* and *Bacteriodes vulgatus* (25). Of note, both *E. Coli* and *B. vulgatus* are also increased in other metabolic-syndrome related diseases and, as such, their abundance parallels the increasing body mass index, hemoglobin A1c levels (26), and insulin resistance (27). Other authors have found that the genera *Ruminococcus* is independently associated with significant fibrosis (24), and, in non-obese MAFLD patients, Ruminococcaceae and Veillonellaceae families were associated with fibrosis severity (28). Interestingly, a recent study proposes an “extra-hepatic” signature that combines the association of muscle fat infiltration (myosteatosis) and a reduction of fecal *Clostridium* sensu stricto for identifying obese patients at higher risk of liver fibrosis (29).

There is, however, substantial heterogeneity and conflicting results among studies aiming to characterize specific signatures associated to the different clinical phenotypes. These discrepancies are probably related to the underlying complex interplay among the different pathogenic elements and the effect of potential confounders (22). For instance, multiple factors such as demographic features (ethnicity, sex, geographical location, etc.), drug consumption, or the circadian oscillations (30) have been shown to impact gut microbiota in terms of proliferation, composition, function and metabolite production, determining the selection of specific genera independently of the disease stage.

Other authors have proposed disease classification signatures based on the metabolomic analysis of substrates derived from gut flora metabolism. Among these products, 3-(4-hydroxyphenyl) lactate, which is derived from aromatic amino acid metabolism, is associated with advanced fibrosis in patients with MAFLD (31). Similarly, circulating levels of trimethylamine N-oxide, which results from choline metabolism, has been shown to correlate with the severity of MAFLD (32).

Experimental and translational studies also support the contribution of small intestine bacterial overgrowth (SIBO) to hepatic steatosis (33). In this regard, patients with MAFLD present an increased prevalence of SIBO, which correlates with the severity of steatosis (34) and hepatic inflammation (35). Of interest, SIBO is associated with higher serum endotoxin levels and higher expression of toll-like receptor 4, CD14 and NFκβ (35), a well-known proinflammatory signaling pathway.

The gut microbiota also plays a critical role as modulator of bile acids (BAs) pool size and composition, by deconjugating primary into secondary BAs. The BAs also regulate microbiome diversity by inducing the production of antimicrobial peptides and modulating the expression of genes related with the innate immunity response, intestinal tight junctions, and lipid metabolism (36). The altered interplay among the circulating BAs and the gut microbiota decreases Farnesoid X receptor (FXR) intestinal signaling, which contributes to the development and progression of the metabolic abnormalities present in MAFLD (37). Conversely, experiments with germ-free mice transplanted with protective gut microbiota against MAFLD, led to increased specific secondary BA, the induction of hepatic BAs transporters, and the repression of hepatic lipogenic genes (38).

Most of the studies targeting the microbiota are focused on the bacterial components, however, fungi and viruses are also present in the intestinal flora. Recently, the analysis of fecal viromes from MAFLD patients has identified a decreased viral diversity compared to healthy volunteers (39). The severity of MAFLD (considered as a NAFLD Activity Score 5-8 or liver cirrhosis) was associated with a significant reduction in the proportion of bacteriophages (viruses that specifically infect and kill bacteria). The addition of viral diversity data to clinical variables in the multivariate analysis, significantly improved model accuracy to identify patients at increased risk of severe disease. The potential infection of the gut by the recently described SARS CoV-2, may exacerbate an existing state of increased intestinal permeability and mucosal inflammation, thereby contributing to the systemic immune dysfunction characteristic of severe COVID-19 (40).

In summary, microbiota dysbiosis and decreased diversity are present in patients with MAFLD as in other metabolic syndrome-related diseases. These alterations are linked to the pathogenesis, but the exact mechanisms underlying disease initiation and progression are partially unknown. Aiming to characterize different MAFLD phenotypes, significant efforts are being made to identify specific microbiome signatures. The multifactorial pathogenesis of the disease hinders these approaches, which, however, may contribute to non-invasive diagnosis, severity stratification, and monitoring in these patients.

### Intestinal Barrier Integrity Regulates Cross-Talk Between the Gut and the Liver

**Figure 1:**
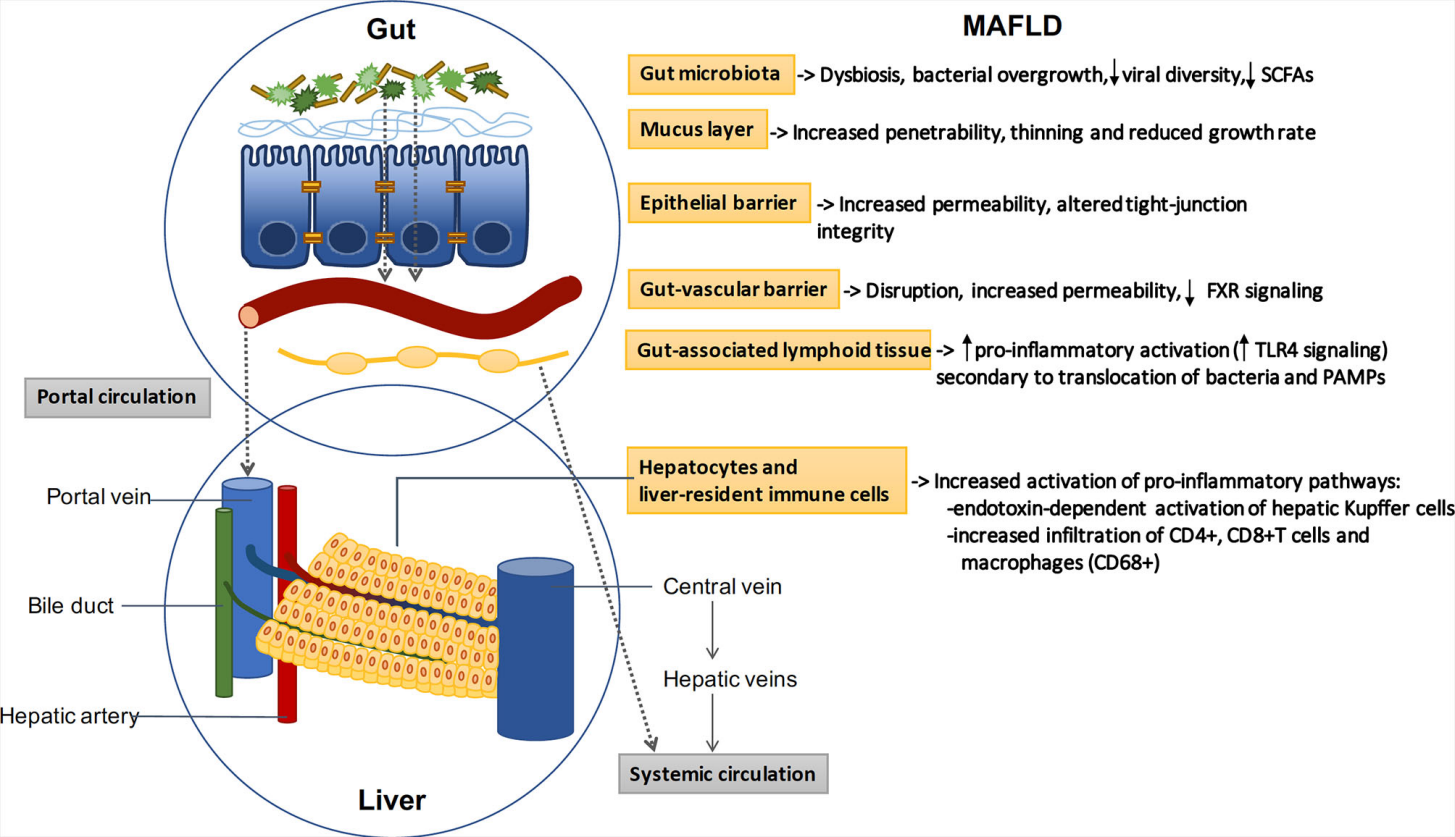
The gut-liver axis: MAFLD induces profound alterations in the gut-liver axis. Beneficial autochthonous bacteria are replaced by potentially pathogenic species leading to dysbiosis and bacterial overgrowth. The increased penetrability of the mucus layer and the increased permeability of the epithelial and vascular barriers allow the translocation of bacteria and related products. Bacterial translocation promotes the activation of gut and liver pro-inflammatory pathways, which play a key role in the pathogenesis of MAFLD. SCFAs: short-chain fatty acids; FXR: Farnesoid X receptor; TLR: toll-like receptor.

The intestinal barrier controls the transport from the gut to the liver and systemic circulation, promoting the absorption of nutrients and water while preventing translocation of pathogens and derived products (pathogen associated molecular patterns or PAMPs) (41). The barrier is composed by a mucus layer and an epithelial monolayer of specialized cells connected by intercellular junctions that seal the space and control the paracellular passage. The mucus prevents the direct contact between the bacteria and the epithelial lining, and is mainly composed by large glycosylated proteins (mucins) secreted by intestinal globet cells (42). In addition to the mucus and the epithelial layer, recent evidence has characterized the gut-vascular barrier, which prevents translocation of bacteria and PAMPs directly into the portal circulation (43) (**Figure 1**).

#### Increased Intestinal Permeability in MAFLD

Increased intestinal permeability has been demonstrated in preclinical models and in patients with MAFLD. Specifically, F11r-knockout mice (a gene which encodes the junctional adhesion molecule A), develop more severe steatohepatitis than control mice following a diet high in saturated fat, fructose and cholesterol (44). In humans, Miele L. et al. investigated intestinal permeability in patients with MAFLD by measuring the urinary excretion of ^51^Cr-ethylene diamine tetraacetate (^51^Cr-EDTA) and analyzing tight junctions integrity by immunohistochemical expression of zona occludens-1 in duodenal biopsy specimens (34). They found that, compared with the values observed in healthy volunteers, ^51^Cr-EDTA excretion was significantly increased, especially in those with moderate/severe steatosis. Further, zonula occludens-1 expression was reduced in intestinal crypts and villi as compared to healthy subjects. Together these data support that disruption of the intercellular tight junctions contributes to increase intestinal permeability and has an important role in the pathogenesis of hepatic steatosis.

Alterations at the mucus layer have been also linked to the pathogenesis of MAFLD. For instance, Mucin-2 deficient mice were protected from high-fat diet induced weight gain, fatty liver, and insulin resistance (45). On the other hand, decreased abundance of *Akkermansia muciniphila*, a mucin-degrading bacteria which represents more than 1% of the microbial community in healthy humans (46), has been associated with thinning of the mucus layer, and a subsequent increase in gut permeability and inflammation (47). Depletion of this gram negative bacteria has been linked to obesity, insulin resistance, hypertension and liver inflammation (48, 49). In line with this findings, oral supplementation with *A. muciniphila* in overweight/obese insulin-resistant volunteers, improved insulin sensitivity, and reduced insulinemia, total cholesterol, markers of liver dysfunction and inflammation (50). On addition, dietary emulsifiers such as carboxymethylcellulose and polysorbate-80 are widely used as components of processed foods. These additives induce a reduction of mucus thickness, which leads to a higher contact of bacteria with the epithelium, and an increased permeability to dextrans, contributing to low grade inflammation and metabolic syndrome (51).

#### Diet Impacts Intestinal Barrier Integrity by Modulating Gut Microbiota

Microbial fermentation of dietary indigestible fibers produces short-chain fatty acids (SCFAs), mainly, acetate, propionate, and butyrate (52). SCFAs serve as energy substrates to the colonic epithelium, modulate cell functions such as histone deacetylation (53), and contribute to control gut inflammation (54), immune homeostasis (55) and appetite (56). Western-style diet (WSD) is rich in saturated fats and simple carbohydrates but poor in dietary fiber, and thus, lower concentrations of SCFAs are found in mice fed with WSD (57). These mice also showed functional defects in the intestinal barrier, specifically, an increased mucus penetrability and a reduced growth rate of the inner mucus layer. Both defects were prevented by fecal microbiota transplantation from chow-fed donors. Of note, administration of *Bifidobacterium longum* restored mucus growth, but not the increased penetrability, which was instead prevented by fiber supplementation (57). Likewise, microbiota transplantation and supplementation with Quercetin (a flavonoid with antioxidant and anti-inflammatory properties) restored SCFAs production in mice fed with high-fat diet (58, 59). Quercetin and other prebiotic compounds, such as polyphenol-rich extracts or some dietary fibers as inulin, may also counteract lipid metabolism dysregulation, and liver and systemic inflammation in preclinical models (60–62). In addition, probiotics, such as wild rice, which is a genus of grasses (Zizania), have shown to reduce body weight, liver steatosis and inflammation in high-fat diet-fed mice (63).

The above preclinical evidences are in line with clinical studies in patients with type 2 diabetes mellitus who received high-fiber diet vs standard of care. Increased availability of non-digestible but fermentable carbohydrates was sufficient to induce a clinically relevant improvement in hemoglobin A1c levels. Fiber supplementation also induced selective promotion of SCFA-producing bacterial strains. This coincided with a significant increase in postprandial glucagon-like peptide-1 and higher levels of fasting peptide YY, which favors insulin secretion and glucose regulation (64). Vitamin D supplementation, that has shown potential anti-fibrotic, anti-inflammatory, and insulin-sensitizing properties, is under investigation following conflicting results about its beneficial effects in MAFLD (65).

All these evidences indicate that the alterations in the intestinal barrier are potentially reversible by modifying the composition of the microbiota with fecal transplantation or dietary approaches.

#### Emerging Data on Gut Vascular Barrier Contribution to MAFLD

The role of the gut vascular barrier in the pathogenesis of MAFLD has also been explored. Data from patients with celiac disease and altered serum transaminases suggest that the disruption of the endothelial barrier may also contribute to liver inflammation and damage (43). Recently, studies have found that high-fat diet modifies the microbiota leading to an early disruption of the epithelial barrier and an increased vascular permeability. The disruption of the endothelial barrier induced by dysbiosis allows bacterial translocation to the liver, and has been found to be an early and required event in MAFLD development (66). Mechanistically, the alteration of the vascular barrier involved the WNT/b-catenin signaling pathway, and, consistent with these findings, obeticholic acid, a FXR receptor agonist that drives b-catenin activation in endothelial cells, protected against barrier disruption and limited steatohepatitis development in mice fed with a high-fat diet. This suggests that protection against vascular barrier disruption could be an additional mechanism by which obeticholic acid may improve outcomes in MALFD.

### The Role of the Immune System in the Pathogenesis of MAFLD

The immune system plays a critical role in the homeostasis and regulation of the gut-liver axis. The accurate balance between immune activation and tolerance is controlled by a network of innate and adaptive immune cells present not only in the liver, but also in the intestine and the adipose tissue (67). The first defense against intestinal pathogens is the gut-associated lymphoid tissue (GALT), which is composed by intraepithelial lymphocytes, Peyer’s patches, the mesenteric lymph nodes and the immune effector cells within the lamina propria (68). The GALT also extends to the large intestine which contains cryptopatches and isolated lymphoid follicles. On the other hand, the liver displays a number of specialized innate and adaptive immune cells responsible for detecting and clearing pathogens that reach the sinusoids. Liver-resident immune cells include antigen presenting cells (APCs) (Kupffer, dendritic and liver sinusoidal endothelial cells), T cells, B cells, natural killer cells and monocytes (69). In addition, the hepatocytes produce essential immune components, and may also act as APCs by expressing MHC-I and II and costimulatory molecules (70).

Despite the continuous challenge, in basal conditions, the gut and the liver immune system act coordinately screening and classifying gut-derived antigens as harmless dietary elements or bacterial pathogens, balancing tolerance and immune activation. However, specific pro-inflammatory pathways are triggered in patients with MAFLD (71). In particular, TLR4 signaling has been identified as a key driver of inflammation and steatohepatitis in diverse experimental animal models of MAFLD (72, 73). On the other hand, NLRP3 inflammasome deficiency attenuates dysbiosis, systemic inflammation and the total cholesterol increase induced in mice exposed to high fat diet (74). Fructose-induced MAFLD is also associated with intestinal bacterial overgrowth and increased intestinal permeability, subsequently leading to an endotoxin-dependent activation of hepatic Kupffer cells (75). Additionally, increased visceral adipose tissue is infiltrated by pro-inflammatory macrophages that induce the release of chemokines and cytokines involved in liver inflammation and hepatic insulin resistance (76).

Immune mechanisms have also been involved in the progression from steatosis to fibrosis. Emerging data highlight the important role of CD4+memory T cells, specifically IL-17A-secreting Th17 and IFNγ-secreting Th1 subsets. Humanized mouse models of diet-induced MAFLD showed a remarkable liver infiltration of CD4+, CD8+T cells and macrophages (CD68+), which were largely confined to fibrotic regions. Conversely, depletion of CD4+T cells abrogated diet-induced inflammatory response and liver fibrosis development (77). Further studies have shown that blocking integrin receptor α4β7-mediated recruitment of CD4+ T cells to the intestine and liver, not only attenuates hepatic inflammation and fibrosis, but also improves metabolic alterations associated with MAFLD (78).

### Therapeutic Interventions Targeting the Gut-Liver-Axis in Patients With MAFLD

Taken together, all the above evidences show that disruption of the gut-liver-axis contributes to the pathogenesis of MAFLD, and thus, there is a great interest in the modulation of their components for therapeutic purposes. These strategies have shown promising results in preclinical studies, however, evidence in human clinical trials is limited so far. Below we have selected emerging therapeutic approaches targeting the gut-liver axis on MAFLD, whose results highlight the complex pathophysiology underlying the disease (**Table 1**).

**Table 1:** Selected therapeutic interventions targeting the gut-liver axis.

Study	Identification	Main results
Safety and efficacy of hydrothermal duodenal mucosal resurfacing in patients with type 2 diabetes: the randomized, double-blind, sham-controlled, multicenter REVITA-2 feasibility trial	NCT02879383	DMR is safe and exerts beneficial disease-modifying metabolic effects in T2D with or without non-alcoholic liver disease (Phase 2)
Effect of duodenal mucosal resurfacing in the treatment of NASH (DMR_NASH_001)	NCT03536650	Completed, no results posted yet
Cenicriviroc treatment for adults with nonalcoholic steatohepatitis and fibrosis: final analysis of the phase 2b CENTAUR study	NCT02217475	A similar proportion on CVC or placebo achieved ≥1-stage fibrosis improvement and no worsening of NASH (Phase 2b)
Cenicriviroc for the treatment of liver fibrosis in adults with nonalcoholic steatohepatitis: AURORA phase 3 study design	NCT03028740	Recruiting (Phase 3)
The fatty acid-bile acid conjugate aramchol reduces liver fat content in patients with nonalcoholic fatty liver disease	NCT01094158	Aramchol is safe, tolerable, and significantly reduces liver fat content in patients with NAFLD (Phase 2)
A Phase 2b, randomized, double-blind, placebo-controlled study evaluating the safety and efficacy of efruxifermin in non-cirrhotic subjects with nonalcoholic steatohepatitis	NCT04767529	Recruiting. Preliminary results indicate a high percentage of fibrosis resolution (Phase 2)
Resmetirom (MGL-3196) for the treatment of non-alcoholic steatohepatitis: a multicenter, randomized, double-blind, placebo-controlled, phase 2 trial	NCT02912260	Significant reduction in hepatic fat in patients with NASH (Phase 2)
Investigation of efficacy and safety of three dose levels of subcutaneous semaglutide once daily versus placebo in subjects with non-alcoholic steatohepatitis.	NCT02970942	Semaglutide resulted in a significantly higher percentage of patients with NASH resolution than placebo. It did not show a significant difference in fibrosis stage (Phase 2)
Researching an effect of GLP-1 agonist on liver steatosis (REALIST)	NCT03648554	Completed, no results posted yet (Phase 4)
Randomized global phase 3 study to evaluate the impact on NASH with fibrosis of obeticholic acid treatment (REGENERATE)	NCT02548351	Interim analysis showed that obeticholic acid 25 mg significantly improved fibrosis and key components of NASH (Phase 3)
Liraglutide safety and efficacy in patients with non-alcoholic steatohepatitis (LEAN): a multicenter, double-blind, randomized, placebo-controlled phase 2 study	NCT01237119	Liraglutide was safe, well tolerated, and led to histological resolution of NASH (Phase 2)
Safety and tolerability of yaq-001 in patients with non-alcoholic steatohepatitis	NCT03962608	Not yet recruiting
The effect of consecutive fecal microbiota transplantation on non-alcoholic fatty liver disease	NCT04465032	Recruiting (Phase 4)
The effect of probiotics on the clinical outcomes and gut microenvironment in patients with fatty liver	NCT04074889	Recruiting
An adaptive design study for the assessment of the safety, tolerability, and pharmacokinetics of RYI-018 (Anti-CB1 monoclonal antibody) after repeat dosing in subjects with non-alcoholic fatty liver disease	NCT03261739	Completed, no results posted yet (Phase 1)
Polyunsaturated fatty acids in patients with NAFLD	NCT02647294	Completed, no results posted yet
An investigator initiated prospective, four arms randomized comparative study of efficacy and safety of saroglitazar, vitamin e and life style modification in patients with nonalcoholic fatty liver disease/non-alcoholic steatohepatitis	NCT04193982	Recruiting
A double-blind, randomized, placebo-controlled, phase 2 study to explore the efficacy and safety of elobixibat in adults with nonalcoholic fatty liver disease or nonalcoholic steatohepatitis	NCT04006145	Completed, no results posted yet (Phase 2)
Effect of silymarin in patients with non-alcoholic fatty liver disease	NCT03749070	Recruiting
Study to assess the efficacy, safety and tolerability of LCQ908 (Pradigastat) in NAFLD patients	NCT01811472	Completed. Preliminary results suggest that LCQ908 was effective, safe and well tolerated (Phase 2)

DMR: duodenal mucosal resurfacing; T2D: type 2 diabetes; NASH: non-alcoholic steatohepatitis; CVC: cenicriviroc; NAFLD: non-alcoholic fatty liver disease; GLP-1: Glucagon-like peptide 1.

Fecal microbiota transplantation (FMT) can potentially restore microbial diversity and function. Therefore, Craven L. and colleagues hypothesized that fecal transplantation from healthy lean donors to MAFLD patients could have metabolic benefits by restoring the integrity of the intestinal barrier. Allogenic FMT failed to decrease insulin resistance or the percentage of hepatic fat assessed by magnetic resonance, however, it did improve intestinal permeability evaluated by the lactulose/mannitol ratio urine test (79). These results are in contrast with the findings of Vrieze et al. that found that infusion of microbiota from lean donors, increased insulin sensitivity in recipients with metabolic syndrome (80). Another interesting study has demonstrated that FMT from metabolically compromised obese donors temporarily worsens insulin sensitivity in recipients with metabolic syndrome, whereas a non-significant increase in insulin sensitivity was observed in recipients of FMT from healthy postgastric bypass donors (81). These results suggest that the response to FMT is modulated by differences in fecal microbiota composition and could explain in part the heterogeneity of the results obtained so far.

Non-absorbable carbons of controlled porosity (Yaq-001) have shown to selectively modulate the composition and function of stool microbiome (82), and thus, safety and tolerability of Yaq-001 is currently under investigation (NCT03962608). Other ongoing study aims to assess the feasibility and safety of endoscopic duodenal mucosal resurfacing in this population (NCT03536650), which can potentially mimic the beneficial metabolic effects on glucose metabolism achieved by surgical upper-intestinal bypass (83). Other strategies aiming to improve insulin resistance and metabolic dysfunction by Glucagon-like peptide-1 analogues have shown positive results in terms of steatohepatitis resolution (84, 85), however, no significant differences in fibrosis regression have yet been achieved. In this regard, an interim analysis from a multicenter, randomized, placebo-controlled trial with obeticholic acid has demonstrated to be the only strategy that significantly improves fibrosis as well as other key components of MAFLD, and warrants further evaluation (86).

## Conclusions

MAFLD is the most prevalent liver disease worldwide. The disruption of the gut-liver axis drives its pathogenesis and progression. Dietary factors may induce profound qualitative and quantitative changes in the gut microbiota, which subsequently impairs the integrity of the epithelial and vascular barriers. The increased translocation of bacteria and PAMPs induce a persistent immune activation that promotes gut and liver inflammation. A better understanding of the different mechanisms involved in the pathogenesis of MAFLD will contribute to develop better diagnostic and therapeutic approaches.

## Author Contributions

RM drafted the manuscript. RM and AA: critical revision for important intellectual content. All authors contributed to the article and approved the submitted version.

## Funding

This work was financed by grants from the Spanish Ministerio de Ciencia e Innovación (SAF 2017-86343-R, and Instituto de Salud Carlos III PI20/01302 awarded to A.A). CIBEREHD is funded by the Instituto de Salud Carlos III. Grants cofinanced by the European Development Regional Fund “A way to achieve Europe” (EDRF).

## Conflict of Interest

The authors declare that the research was conducted in the absence of any commercial or financial relationships that could be construed as a potential conflict of interest.

The reviewer JG-G declared a shared affiliation with the authors to the handling editor at the time of review.

## References

[1] Younossi ZM Koenig AB Abdelatif D Fazel Y Henry L Wymer M. Global epidemiology of nonalcoholic fatty liver disease—Meta-analytic assessment of prevalence, incidence, and outcomes. Hepatology (2016) 64:73–84. 10.1002/hep.28431 26707365

[2] Estes C Razavi H Loomba R Younossi Z Sanyal AJ. Modeling the epidemic of nonalcoholic fatty liver disease demonstrates an exponential increase in burden of disease. Hepatology (2018) 67:123–33. 10.1002/hep.29466 PMC5767767 28802062

[3] Angulo P Kleiner DE Dam-Larsen S Adams LA Bjornsson ES Charatcharoenwitthaya P. Liver fibrosis, but no other histologic features, is associated with long-term outcomes of patients with nonalcoholic fatty liver disease. Gastroenterology (2015) 149:389–97. 10.1053/j.gastro.2015.04.043 PMC4516664 25935633

[4] Eslam M Newsome PN Sarin SK Anstee QM Targher G Romero-Gomez M. A new definition for metabolic dysfunction-associated fatty liver disease: An international expert consensus statement. J Hepatol (2020) 73:202–9. 10.1016/j.jhep.2020.03.039 32278004

[5] Albillos A de Gottardi A Rescigno M. The gut-liver axis in liver disease: Pathophysiological basis for therapy. J Hepatol (2020) 72:558–77. 10.1016/j.jhep.2019.10.003 31622696

[6] Leung C Rivera L Furness JB Angus PW. The role of the gut microbiota in NAFLD. Nat Rev Gastroenterol Hepatol (2016) 13:412–25. 10.1038/nrgastro.2016.85 27273168

[7] Parthasarathy G Revelo X Malhi H. Pathogenesis of nonalcoholic steatohepatitis: an overview. Hepatol Commun (2020) 4:478–92. 10.1002/hep4.1479 PMC7109346 32258944

[8] Wiest R Albillos A Trauner M Bajaj JS Jalan R. Targeting the gut-liver axis in liver disease. J Hepatol (2017) 67:1084–103. 10.1016/j.jhep.2017.05.007 28526488

[9] Albillos A Lario M Álvarez-Mon M. Cirrhosis-associated immune dysfunction: distinctive features and clinical relevance. J Hepatol (2014) 61:1385–96. 10.1016/j.jhep.2014.08.010 25135860

[10] Guarner F Malagelada J-R. Gut flora in health and disease. Lancet (2003) 361:512–9. 10.1016/S0140-6736(03)12489-0 12583961

[11] Konjevod M Nikolac Perkovic M Sáiz J Svob Strac D Barbas C Rojo D. Metabolomics analysis of microbiota-gut-brain axis in neurodegenerative and psychiatric diseases. J Pharm BioMed Anal (2020) 194:113681. 10.1016/j.jpba.2020.113681 33279302

[12] Docimo G Cangiano A Romano RM Pignatelli MF Offi C Paglionico VA. The human microbiota in endocrinology: implications for pathophysiology, treatment, and prognosis in thyroid diseases. Front Endocrinol (Lausanne) (2020) 11:586529. 10.3389/fendo.2020.586529 33343507 PMC7746874

[13] Cheng Y Ling Z Li L. The intestinal microbiota and colorectal cancer. Front Immunol (2020) 11:615056. 10.3389/fimmu.2020.615056 33329610 PMC7734048

[14] Tilg H Zmora N Adolph TE Elinav E. The intestinal microbiota fuelling metabolic inflammation. Nat Rev Immunol (2020) 20:40–54. 10.1038/s41577-019-0198-4 31388093

[15] Turnbaugh PJ Ley RE Mahowald MA Magrini V Mardis ER Gordon JI. An obesity-associated gut microbiome with increased capacity for energy harvest. Nature (2006) 444:1027–31. 10.1038/nature05414 17183312

[16] Le Chatelier E Nielsen T Qin J Prifti E Hildebrand F Falony G. Richness of human gut microbiome correlates with metabolic markers. Nature (2013) 500:541–6. 10.1038/nature12506 23985870

[17] Qin J Li Y Cai Z Li S Zhu J Zhang F. A metagenome-wide association study of gut microbiota in type 2 diabetes. Nature (2012) 490:55–60. 10.1038/nature11450 23023125

[18] Turnbaugh PJ Hamady M Yatsunenko T Cantarel BL Duncan A Ley RE. A core gut microbiome in obese and lean twins. Nature (2009) 457:480–4. 10.1038/nature07540 PMC2677729 19043404

[19] Sharpton SR Schnabl B Knight R Loomba R. Current concepts, opportunities, and challenges of gut microbiome-based personalized medicine in nonalcoholic fatty liver disease. Cell Metab (2021) 33:21–32. 10.1016/j.cmet.2020.11.010 33296678 PMC8414992

[20] Bäckhed F Ding H Wang T Hooper LV Gou YK Nagy A. The gut microbiota as an environmental factor that regulates fat storage. Proc Natl Acad Sci USA (2004) 101:15718–23. 10.1073/pnas.0407076101 PMC524219 15505215

[21] Le Roy T Llopis M Lepage P Bruneau A Rabot S Bevilacqua C. Intestinal microbiota determines development of non-alcoholic fatty liver disease in mice. Gut (2013) 62:1787–94. 10.1136/gutjnl-2012-303816 23197411

[22] Aron-Wisnewsky J Vigliotti C Witjes J Le P Holleboom AG Verheij J. Gut microbiota and human NAFLD: disentangling microbial signatures from metabolic disorders. Nat Rev Gastroenterol Hepatol (2020) 17:279–97. 10.1038/s41575-020-0269-9 32152478

[23] Shen F Zheng R-D Sun X-Q Ding W-J Wang X-Y Fan J-G. Gut microbiota dysbiosis in patients with non-alcoholic fatty liver disease. Hepatobiliary Pancreat Dis Int (2017) 16:375–81. 10.1016/S1499-3872(17)60019-5 28823367

[24] Boursier J Mueller O Barret M Machado M Fizanne L Araujo-Perez F. The severity of nonalcoholic fatty liver disease is associated with gut dysbiosis and shift in the metabolic function of the gut microbiota. Hepatology (2016) 63:764–75. 10.1002/hep.28356 PMC4975935 26600078

[25] Loomba R Seguritan V Li W Long T Klitgord N Bhatt A. Gut microbiome-based metagenomic signature for non-invasive detection of advanced fibrosis in human nonalcoholic fatty liver disease. Cell Metab (2017) 25:1054–62. 10.1016/j.cmet.2017.04.001 PMC5502730 28467925

[26] Aron-Wisnewsky J Prifti E Belda E Ichou F Kayser BD Dao MC. Major microbiota dysbiosis in severe obesity: fate after bariatric surgery. Gut (2019) 68:70–82. 10.1136/gutjnl-2018-316103 29899081 PMC7143256

[27] Pedersen HK Gudmundsdottir V Nielsen HB Hyotylainen T Nielsen T Jensen BAH. Human gut microbes impact host serum metabolome and insulin sensitivity. Nature (2016) 535:376–81. 10.1038/nature18646 27409811

[28] Lee G You HJ Bajaj JS Joo SK Yu J Park S. Distinct signatures of gut microbiome and metabolites associated with significant fibrosis in non-obese NAFLD. Nat Commun (2020) 11:4982. 10.1038/s41467-020-18754-5 33020474 PMC7536225

[29] Lanthier N Rodriguez J Nachit M Hiel S Trefois P Neyrinck AM. Microbiota analysis and transient elastography reveal new extra-hepatic components of liver steatosis and fibrosis in obese patients. Sci Rep (2021) 11:1–14. 10.1038/s41598-020-79718-9 33436764 PMC7804131

[30] Thaiss CA Levy M Korem T Dohnalová L Shapiro H Jaitin DA. Microbiota diurnal rhythmicity programs host transcriptome oscillations. Cell (2016) 167:1495–510. 10.1016/j.cell.2016.11.003 27912059

[31] Caussy C Hsu C Lo M-T Liu A Bettencourt R Ajmera VH. Link between gut-microbiome derived metabolite and shared gene-effects with hepatic steatosis and fibrosis in NAFLD. Hepatology (2018) 68:918–32. 10.1002/hep.29892 PMC6151296 29572891

[32] Chen Y Liu Y Zhou R Chen X Wang C Tan X. Associations of gut-flora-dependent metabolite trimethylamine-N-oxide, betaine and choline with non-alcoholic fatty liver disease in adults. Sci Rep (2016) 6:19076. 10.1038/srep19076 26743949 PMC4705470

[33] Wijarnpreecha K Lou S Watthanasuntorn K Kroner PT Cheungpasitporn W Lukens FJ. Small intestinal bacterial overgrowth and nonalcoholic fatty liver disease: a systematic review and meta-analysis. Eur J Gastroenterol Hepatol (2020) 32:601–8. 10.1097/MEG.0000000000001541 31567712

[34] Miele L Valenza V La Torre G Montalto M Cammarota G Ricci R. Increased intestinal permeability and tight junction alterations in nonalcoholic fatty liver disease. Hepatology (2009) 49:1877–87. 10.1002/hep.22848 19291785

[35] Kapil S Duseja A Sharma BK Singla B Chakraborti A Das A. Small intestinal bacterial overgrowth and toll-like receptor signaling in patients with non-alcoholic fatty liver disease. J Gastroenterol Hepatol (2016) 31:213–21. 10.1111/jgh.13058 26212089

[36] Wang W Zhao J Gui W Sun D Dai H Xiao L. Tauroursodeoxycholic acid inhibits intestinal inflammation and barrier disruption in mice with non-alcoholic fatty liver disease. Br J Pharmacol (2018) 175:469–84. 10.1111/bph.14095 PMC5773980 29139555

[37] Chen J Vitetta L. Gut microbiota metabolites in nafld pathogenesis and therapeutic implications. Int J Mol Sci (2020) 21:1–19. 10.3390/ijms21155214 PMC7432372 32717871

[38] Petrov PD García-Mediavilla MV Guzmán C Porras D Nistal E Martínez-Flórez S. A network involving gut microbiota, circulating bile acids, and hepatic metabolism genes that protects against non-alcoholic fatty liver disease. Mol Nutr Food Res (2019) 63:1900487. 10.1002/mnfr.201900487 31322321

[39] Lang S Demir M Martin A Jiang L Zhang X Duan Y. Intestinal virome signature associated with severity of nonalcoholic fatty liver disease. Gastroenterology (2020) 159:1839–52. 10.1053/j.gastro.2020.07.005 PMC8404510 32652145

[40] Assante G Williams R Youngson NA. Is the increased risk for MAFLD patients to develop severe COVID-19 linked to perturbation of the gut-liver axis? J Hepatol (2021) 74:487–8. 10.1016/j.jhep.2020.05.051 PMC7305888 32574578

[41] Arab JP Martin-Mateos RM Shah VH. Gut–liver axis, cirrhosis and portal hypertension: the chicken and the egg. Hepatol Int (2018) 12:24–33. 10.1007/s12072-017-9798-x 28550391 PMC6876989

[42] Johansson MEV Sjövall H Hansson GC. The gastrointestinal mucus system in health and disease. Nat Rev Gastroenterol Hepatol (2013) 10:352–61. 10.1038/nrgastro.2013.35 PMC3758667 23478383

[43] Spadoni I Zagato E Bertocchi A Paolinelli R Hot E Di Sabatino A. A gut-vascular barrier controls the systemic dissemination of bacteria. Sci (80-) (2015) 350:830–4. 10.1126/science.aad0135 26564856

[44] Rahman K Desai C Iyer SS Thorn NE Kumar P Liu Y. Loss of junctional adhesion molecule a promotes severe steatohepatitis in mice on a diet high in saturated fat, fructose, and cholesterol. Gastroenterology (2016) 151:733–46. 10.1053/j.gastro.2016.06.022 PMC5037035 27342212

[45] Hartmann P Seebauer CT Mazagova M Horvath A Wang L Llorente C. Deficiency of intestinal mucin-2 protects mice from diet-induced fatty liver disease and obesity. Am J Physiol - Gastrointest Liver Physiol (2016) 310:310–22. 10.1152/ajpgi.00094.2015 PMC4773827 26702135

[46] Derrien M Collado MC Ben-Amor K Salminen S de Vos WM. The mucin degrader akkermansia muciniphila is an abundant resident of the human intestinal tract. Appl Environ Microbiol (2008) 74:1646–8. 10.1128/AEM.01226-07 PMC2258631 18083887

[47] Derrien M Van Baarlen P Hooiveld G Norin E Müller M de Vos WM. Modulation of mucosal immune response, tolerance, and proliferation in mice colonized by the mucin-degrader Akkermansia muciniphila. Front Microbiol (2011) 2:166 10.3389/fmicb.2011.00166 21904534 PMC3153965

[48] Everard A Belzer C Geurts L Ouwerkerk JP Druart C Bindels LB. Cross-talk between Akkermansia muciniphila and intestinal epithelium controls diet-induced obesity. Proc Natl Acad Sci (2013) 110:9066–71. 10.1073/pnas.1219451110 PMC3670398 23671105

[49] Plovier H Everard A Druart C Depommier C Van Hul M Geurts L. A purified membrane protein from Akkermansia muciniphila or the pasteurized bacterium improves metabolism in obese and diabetic mice. Nat Med (2017) 23:107–13. 10.1038/nm.4236 27892954

[50] Depommier C Everard A Druart C Plovier H Van Hul M Vieira-Silva S. Supplementation with Akkermansia muciniphila in overweight and obese human volunteers: a proof-of-concept exploratory study. Nat Med (2019) 25:1096–103. 10.1038/s41591-019-0495-2 PMC6699990 31263284

[51] Chassaing B Koren O Goodrich JK Poole AC Srinivasan S Ley RE. Dietary emulsifiers impact the mouse gut microbiota promoting colitis and metabolic syndrome. Nature (2015) 519:92–6. 10.1038/nature14232 PMC4910713 25731162

[52] Koh A De Vadder F Kovatcheva-Datchary P Bäckhed F. From dietary fiber to host physiology: Short-chain fatty acids as key bacterial metabolites. Cell (2016) 165:1332–45. 10.1016/j.cell.2016.05.041 27259147

[53] Donohoe DR Collins LB Wali A Bigler R Sun W Bultman SJ. The Warburg effect dictates the mechanism of butyrate-mediated histone acetylation and cell proliferation. Mol Cell (2012) 48:612–26. 10.1016/j.molcel.2012.08.033 PMC3513569 23063526

[54] Macia L Tan J Vieira AT Leach K Stanley D Luong S. Metabolite-sensing receptors GPR43 and GPR109A facilitate dietary fibre-induced gut homeostasis through regulation of the inflammasome. Nat Commun (2015) 6:6734. 10.1038/ncomms7734 25828455

[55] Belkaid Y Harrison OJ. Homeostatic immunity and the microbiota. Immunity (2017) 46:562–76. 10.1016/j.immuni.2017.04.008 PMC5604871 28423337

[56] Frost G Sleeth ML Sahuri-Arisoylu M Lizarbe B Cerdan S Brody L. The short-chain fatty acid acetate reduces appetite *via a* central homeostatic mechanism. Nat Commun (2014) 5:3611. 10.1038/ncomms4611 24781306 PMC4015327

[57] Schroeder BO Birchenough GMH Ståhlman M Arike L Johansson MEV Hansson GC. Bifidobacteria or fiber protects against diet-induced microbiota-mediated colonic mucus deterioration. Cell Host Microbe (2018) 23:27–40. 10.1016/j.chom.2017.11.004 29276171 PMC5764785

[58] Porras D Nistal E Martínez-Flórez S Olcoz JL Jover R Jorquera F. Functional interactions between gut microbiota transplantation, quercetin, and high-fat diet determine non-alcoholic fatty liver disease development in germ-free mice. Mol Nutr Food Res (2019) 63:e1800930. 10.1002/mnfr.201800930 30680920

[59] Porras D Nistal E Martínez-Flórez S Pisonero-Vaquero S Olcoz JL Jover R. Protective effect of quercetin on high-fat diet-induced non-alcoholic fatty liver disease in mice is mediated by modulating intestinal microbiota imbalance and related gut-liver axis activation. Free Radic Biol Med (2017) 102:188–202. 10.1016/j.freeradbiomed.2016.11.037 27890642

[60] Yang Z Liu G Wang Y Yin J Wang J Xia B. Fucoidan A2 from the brown seaweed Ascophyllum nodosum lowers lipid by improving reverse cholesterol transport in C57BL/6J mice fed a high-fat diet. J Agric Food Chem (2019) 67:5782–91. 10.1021/acs.jafc.9b01321 31055921

[61] Wu S Hu R Nakano H Chen K Liu M He X. Modulation of gut microbiota by Lonicera caerulea L. Berry polyphenols in a mouse model of fatty liver induced by high fat diet. Molecules (2018) 23:3213. 10.3390/molecules23123213 PMC6321169 30563142

[62] Bao T He F Zhang X Zhu L Wang Z Lu H. Inulin exerts beneficial effects on non-alcoholic fatty liver disease *via* modulating gut microbiome and suppressing the Lipopolysaccharide-toll-like receptor 4-Mψ-nuclear factor-κB-nod-like receptor protein 3 pathway *via* gut-Liver axis in mice. Front Pharmacol (2020) 11:558525. . 10.3389/fphar.2020.558525 33390939 PMC7774311

[63] Hou XD Yan N Du YM Liang H Zhang ZF Yuan XL. Consumption of wild rice (zizania latifolia) prevents metabolic associated fatty liver disease through the modulation of the gut microbiota in mice model. Int J Mol Sci (2020) 21:1–15. 10.3390/ijms21155375 PMC7432455 32751062

[64] Zhao L Zhang F Ding X Wu G Lam YY Wang X. Gut bacteria selectively promoted by dietary fibers alleviate type 2 diabetes. Sci (80-) (2018) 359:1151–6. 10.1126/science.aao5774 29590046

[65] Barchetta I Cimini FA Cavallo MG. Vitamin D and metabolic dysfunction-associated fatty liver disease (MAFLD): An update. Nutrients (2020) 12:3302. 10.3390/nu12113302 PMC7693133 33126575

[66] Mouries J Brescia P Silvestri A Spadoni I Sorribas M Wiest R. Microbiota-driven gut vascular barrier disruption is a prerequisite for non-alcoholic steatohepatitis development. J Hepatol (2019) 71:1216–28. 10.1016/j.jhep.2019.08.005 PMC6880766 31419514

[67] Jenne CN Kubes P. Immune surveillance by the liver. Nat Immunol (2013) 14:996–1006. 10.1038/ni.2691 24048121

[68] Mowat AM. Anatomical basis of tolerance and immunity to intestinal antigens. Nat Rev Immunol (2003) 3:331–41. 10.1038/nri1057 12669023

[69] Zhao J Zhang S Liu Y He X Qu M Xu G. Single-cell RNA sequencing reveals the heterogeneity of liver-resident immune cells in human. Cell Discovery (2020) 6:22. 10.1038/s41421-020-0157-z PMC7186229 32351704

[70] Franco A Barnaba V Natali P Balsano C Musca A Balsano F. Expression of class I and class II major histocompatibility complex antigens on human hepatocytes. Hepatology (1988) 8:449–54. 10.1002/hep.1840080302 2453428

[71] Schuster S Cabrera D Arrese M Feldstein AE. Triggering and resolution of inflammation in NASH. Nat Rev Gastroenterol Hepatol (2018) 15:349–64. 10.1038/s41575-018-0009-6 29740166

[72] Liu J Zhuang Z Bian D Ma X Xun Y Yang W. Toll-like receptor-4 signalling in the progression of non-alcoholic fatty liver disease induced by high-fat and high-fructose diet in mice. Clin Exp Pharmacol Physiol (2014) 41:482–8. 10.1111/1440-1681.12241 24739055

[73] Longo L Tonin Ferrari J Rampelotto PH Hirata Dellavia G Pasqualotto A P Oliveira C. Gut dysbiosis and increased intestinal permeability drive microRNAs, NLRP-3 inflammasome and liver fibrosis in a nutritional model of non-alcoholic steatohepatitis in adult male sprague dawley rats. Clin Exp Gastroenterol (2020) 13:351–68. 10.2147/CEG.S262879 PMC7509481 32982365

[74] Sokolova M Yang K Hansen SH Louwe MC Kummen M Hov JER. NLRP3 inflammasome deficiency attenuates metabolic disturbances involving alterations in the gut microbial profile in mice exposed to high fat diet. Sci Rep (2020) 10:1–16. 10.1038/s41598-020-76497-1 33273482 PMC7712828

[75] Spruss A Kanuri G Wagnerberger S Haub S Bischoff SC Bergheim I. Toll-like receptor 4 is involved in the development of fructose-induced hepatic steatosis in mice. Hepatology (2009) 50:1094–104. 10.1002/hep.23122 19637282

[76] Stanton MC Chen S-C Jackson JV Rojas-Triana A Kinsley D Cui L. Inflammatory signals shift from adipose to liver during high fat feeding and influence the development of steatohepatitis in mice. J Inflammation (2011) 8:8. 10.1186/1476-9255-8-8 PMC3070617 21410952

[77] Her Z Tan JHL Lim Y-S Tan SY Chan XY Tan WWS. CD4+ T cells mediate the development of liver fibrosis in high fat diet-induced NAFLD in humanized mice. Front Immunol (2020) 11:580968. 10.3389/fimmu.2020.580968 33013934 PMC7516019

[78] Rai RP Liu Y Iyer SS Liu S Gupta B Desai C. Blocking integrin α4β7-mediated CD4 T cell recruitment to the intestine and liver protects mice from western diet-induced non-alcoholic steatohepatitis. J Hepatol (2020) 73:1013–22. 10.1016/j.jhep.2020.05.047 PMC7839272 32540177

[79] Craven L Rahman A Nair Parvathy S Beaton M Silverman J Qumosani K. Allogenic fecal microbiota transplantation in patients with nonalcoholic fatty liver disease improves abnormal small intestinal permeability. Am J Gastroenterol (2020) 115:1055–65. 10.14309/ajg.0000000000000661 32618656

[80] Vrieze A Van Nood E Holleman F Salojärvi J Kootte RS Bartelsman JFWM. Transfer of intestinal microbiota from lean donors increases insulin sensitivity in individuals with metabolic syndrome. Gastroenterology (2012) 143:913–6. 10.1053/j.gastro.2012.06.031 22728514

[81] de Groot P Scheithauer T Bakker guido J Prodan A Levin E Khan MT. Donor metabolic characteristics drive effects of faecal microbiota transplantation on recipient insulin sensitivity, energy expenditure and intestinal transit time. Gut (2020) 69:502–12. 10.1136/gutjnl-2019-318320 PMC7034343 31147381

[82] Macnaughtan J Ranchal I Soeda J Sawhney R Oben J Davies N. Oral therapy with non-absorbable carbons of controlled porosity (YAQ-001) selectively modulates stool microbiome and its function and this is associated with restoration of immune function and inflammasome activation. J Hepatol (2015) 62:S240. 10.1016/S0168-8278(15)30110-0

[83] Zervos EE Agle SC Warren AJ Lang CG Fitzgerald TL Dar M. Amelioration of insulin requirement in patients undergoing duodenal bypass for reasons other than obesity implicates foregut factors in the pathophysiology of type II diabetes. J Am Coll Surg (2010) 210:564–72. 10.1016/j.jamcollsurg.2009.12.025 20421005

[84] Newsome PN Buchholtz K Cusi K Linder M Okanoue T Ratziu V. NN9931-4296 Investigators. A placebo-controlled trial of subcutaneous semaglutide in nonalcoholic steatohepatitis. N Engl J Med (2021) 384:1113–24. 10.1056/NEJMoa2028395 33185364

[85] Armstrong MJ Gaunt P Aithal GP Barton D Hull D Parker R. Liraglutide safety and efficacy in patients with non-alcoholic steatohepatitis (LEAN): a multicentre, double-blind, randomised, placebo-controlled phase 2 study. Lancet (2016) 387:679–90. 10.1016/S0140-6736(15)00803-X 26608256

[86] Younossi ZM Ratziu V Loomba R Rinella M Anstee QM Goodman Z. Obeticholic acid for the treatment of non-alcoholic steatohepatitis: interim analysis from a multicentre, randomised, placebo-controlled phase 3 trial. Lancet (2019) 394:2184–96. 10.1016/S0140-6736(19)33041-7 31813633

